# HIV Viremia and T-Cell Activation Differentially Affect the Performance of Glomerular Filtration Rate Equations Based on Creatinine and Cystatin C

**DOI:** 10.1371/journal.pone.0082028

**Published:** 2013-12-23

**Authors:** Bhavna Bhasin, Bryan Lau, Mohamed G. Atta, Derek M. Fine, Michelle M. Estrella, George J. Schwartz, Gregory M. Lucas

**Affiliations:** 1 Department of Medicine, Johns Hopkins University School of Medicine, Baltimore, Maryland, United States of America; 2 Department of Epidemiology, Johns Hopkins University Bloomberg School of Public Health, Baltimore, Maryland, United States of America; 3 Department of Pediatrics, University of Rochester School of Medicine, Rochester, New York, United States of America; South Texas Veterans Health Care System and University Health Science Center San Antonio, United States of America

## Abstract

**Background:**

Serum creatinine and cystatin C are used as markers of glomerular filtration rate (GFR). The performance of these GFR markers relative to exogenously measured GFR (mGFR) in HIV-positive individuals is not well established.

**Methods:**

We assessed the performance of the chronic kidney disease epidemiology collaboration equations based on serum concentrations of creatinine (eGFR_cr_), cystatin C (eGFR_cys_) and both biomarkers combined (eGFR_cr-cys_) in 187 HIV-positive and 98 HIV-negative participants. Measured GFR was calculated by plasma iohexol clearance. Bias and accuracy were defined as the difference between eGFR and mGFR and the percentage of eGFR observations within 30% of mGFR, respectively. Activated CD4 and CD8 T-cells (CD38+ HLA-DR+) were measured by flow cytometry.

**Results:**

The median mGFR was >100 ml/min/1.73 m^2^ in both groups. All equations tended to be less accurate in HIV-positive than in HIV-negative subjects, with eGFR_cr-cys_ being the most accurate overall. In the HIV-positive group, eGFR_cys_ was significantly less accurate and more biased than eGFR_cr_ and eGFR_cr_cys_. Additionally eGFR_cys_ bias and accuracy were strongly associated with use of antiretroviral therapy, HIV RNA suppression, and percentages of activated CD4 or CD8 T-cells. Hepatitis C seropositivity was associated with larger eGFR_cys_ bias in both HIV-positive and HIV-negative groups. In contrast, eGFR_cr_ accuracy and bias were not associated with HIV-related factors, T-cell activation, or hepatitis C.

**Conclusions:**

The performance of eGFR_cys_ relative to mGFR was strongly correlated with HIV treatment factors and markers of T-cell activation, which may limit its usefulness as a GFR marker in this population.

## Introduction

Early detection of renal disease is important in HIV-positive individuals to implement appropriate interventions and remove potentially nephrotoxic drugs. In clinical practice, serum creatinine is widely used as an intrinsic glomerular filtration rate (GFR) marker. Cystatin C, a constitutively-produced cysteine proteinase, has been proposed as an alternative and potentially superior GFR marker [1], [2]. However, cystatin C concentrations may be affected by inflammation [3], which could be relevant in HIV-infected persons. The Chronic Kidney Disease Epidemiology Collaboration (CKD-EPI) equations (one based on creatinine, one based on cystatin C, and one based on both biomarkers) have been shown to be more accurate than the Modification of Diet in Renal Disease (MDRD) equation (which uses creatinine), particularly in persons with GFR >60 ml/min/1.73 m^2^ [4], [5].

Two recent studies that measured GFR with an exogenous marker in HIV-infected individuals found no evidence that cystatin C-based estimates were more accurate or precise than creatinine-based estimates [6], [7]. Additional data are needed to elucidate the strengths and limitations of GFR equations based on creatinine, cystatin C, or both biomarkers in this population, considering HIV-related immune activation. Using iohexol clearance from plasma to exogenously measure GFR, we assessed the performance of the CKD-EPI equations and clinical factors affecting performance, in HIV-positive participants and a demographically similar HIV-negative comparison group.

## Methods

### Study design and population

We recruited HIV-positive subjects from the Johns Hopkins HIV Clinic and HIV-negative subjects from the community and from the AIDS Link to IntraVenous Experience (ALIVE) cohort [8], the latter to oversample HIV-negative individuals with a history of injection drug use and hepatitis C infection. Participants were screened for eligibility at two screening visits. Inclusion criteria were age 18 years or older and estimated GFR ≥60 ml/min/1.73 m^2^ (by MDRD equation [9]), the latter because the primary objective of the cohort was to assess measured GFR change over time in subjects with initially normal estimated kidney function. Exclusion criteria included history of radiocontrast allergy, pregnancy, diabetes mellitus (history of diabetes diagnosis or treatment for diabetes, or random serum glucose >130 mg/dL or glycosylated hemoglobin >6.5% at screening), uncontrolled hypertension (systolic blood pressure >160 mm Hg or diastolic blood pressure >100 mm Hg), collagen vascular disease, and severe or life-threatening comorbid conditions.

### Ethics statement

Participants provided written informed consent and the study was approved by the Johns Hopkins Medicine Institutional Review Board.

### Data collection and laboratory measurements

We collected demographic, behavioral, and pharmacologic data by interview and medical record review, and measured height, weight, and blood pressure. Laboratory data included hepatitis C serostatus and plasma concentrations of creatinine, cystatin C, and high-sensitivity C-reactive protein (hsCRP). Creatinine was measured with an enzymatic assay (Creatinine Plus, Roche Diagnostics, Basel, Switzerland) that was traceable to an isotope dilution mass spectrometry reference method [10]. Cystatin C was measured using a particle enhanced turbidimetric immunoassay (Gentian AS, Norway), with values standardized to a certified reference material from the Institute for Reference Materials and Measurements [11]. We used flow cytometry to measure the percentages of activated CD4 and CD8 cells, defined by the presence of CD38 and HLA-DR surface markers [12], [13]. We also measured urine concentrations of albumin and creatinine. In the HIV-positive group we measured CD4 cell count by flow cytometry and HIV RNA level with Amplicor HIV-1 MONIOTR Test, v1.5 (Roche Molecular Diagnostics), which had a lower limit of detection of 400 copies/mL.

Iohexol clearance was measured by placing two peripheral intravenous catheters and infusing a weighed dose of iohexol (5 mL; GE Healthcare, Amersham Division, Princeton, NJ) into one catheter. Blood samples were drawn at 10, 30, 120, and 240 minutes from the second catheter. Serum iohexol concentrations were measured by high performance liquid chromatography. Measured GFR (mGFR) was calculated using a 2-compartment model described by Schwartz and colleagues [14].

### Definitions and statistical analysis

Estimated GFR was calculated using CKD-EPI equations based on plasma creatinine (eGFR_cr_), cystatin C (eGFR_cys_), or both markers (eGFR_cr-cys_) [15]. We used three parameters to assess the performance of GFR estimating equations relative to mGFR. Accuracy was defined as the percent of eGFR observations within 30% of mGFR, with 95% confidence intervals (CI) determined by exact methods. Supplemental accuracy estimates were calculated for percent of values within 10% of mGFR. Bias was defined as the difference between eGFR and mGFR, where values above and below 0 corresponded to overestimation and underestimation of mGFR, respectively. Precision was defined as the interquartile range (IQR) for bias, with 95% CI determined by bootstrapping.

We compared the accuracy, bias and precision of each estimating equation between the HIV-positive and HIV-negative groups, and compared the performance of the equations with one another, separately in the HIV-positive and HIV-negative groups. We used Fisher's exact and Wilcoxon rank sum tests to compare categorical and continuous variables between groups, respectively. We determined the statistical significance of differences between equations in each group with McNemar's test for accuracy, Wilcoxon sign rank test for bias, and bootstrapping for precision.

We assessed the association of clinical factors with accuracy and bias of the single biomarker equations, eGFR_cr_ and eGFR_cys_. We stratified age, body mass index (BMI), and hsCRP at the median values for the complete study sample, and used group-specific medians for activated CD4 and CD8 cells, due to large differences in these variables between HIV-positive and HIV-negative subjects. There were too few non-African American subjects for meaningful comparisons by race.

Because our analysis included a large number of statistical comparisons, we used the method of Benjamini and Hochberg to reduce the likelihood of making type-1 errors [16]. In this method, the P value thresholds for rejecting null hypotheses are adjusted downward (e.g., <0.05) according to the total number of comparisons and the position of a given test in the ascending rank order of all test-based P values. We adjusted P values to maintain a false discovery rate of 0.05, meaning that of the tests deemed to be statistically significant by the revised P value thresholds, only 5% would be anticipated to be ‘false discoveries’. P value thresholds for baseline comparisons between HIV-positive and HIV-negative subjects (Table 1) were not modified for false discovery. However, in Tables 2,3, 4, actual P values are shown and bold font was used to indicate statistical significance using the false discovery approach.

**Table 1:** Clinical characteristics of HIV-positive and HIV-negative participants.

Clinical characteristics		HIV-positive (n = 187)	HIV-negative (n = 98)	P value
Age, years, median (P_25_, P_75_)		49 (45, 53)	49 (45, 54)	0.58
Body mass index, kg/m^2^, median (P_25_, P_75_)		26 (23, 31)	27 (23, 33)	0.21
Sex	Female, n (%)	66 (35)	18 (18)	0.0027
	Male, n (%)	121 (65)	80 (82)	
Race	White, n (%)	11 (6)	8 (8)	0.46
	Black, n (%)	176 (94)	90 (92)	
Current smoker, n (%)		124 (66)	60 (61)	0.44
History of hypertension, n (%)		65 (35)	21 (21)	0.021
History of cardiovascular disease, n (%)		21 (11)	4 (4)	0.048
Hepatitis C seropositive, n (%)		100 (54)	28 (29)	0.0001
Systolic blood pressure, mm Hg, median (P_25_, P_75_)		120 (108, 131)	126 (113, 135)	0.0074
Diastolic blood pressure, mm Hg, median (P_25_, P_75_)		71 (65, 77)	73 (66, 82)	0.058
Glycosylated hemoglobin, %, median (P_25_, P_75_)		5.4 (5.1, 5.7)	5.5 (5.3, 5.8)	0.038
High-sensitivity C-reactive protein, mg/dL, median (P_25_, P_75_)		1.7 (0.6, 4.2)	1.9 (0.7, 5.5)	0.43
Percentage activated^a^ CD4 cells, median (P_25_, P_75_)		8.3 (5.4, 14.1)	3.8 (3.1–5.9)	<0.0001
Percentage activated^a^ CD8 cells, median (P_25_, P_75_)		30.7 (19.2, 46.9)	10.8 (7.7, 20.5)	<0.0001
Urine albumin-creatinine ratio, mg/g, median (P_25_, P_75_)		7 (3, 19)	5 (3,11)	0.18
Urine albumin-creatinine ratio >30 mg/g, n (%)		36 (19)	9 (9)	0.027
Serum creatinine, mg/dL, median (P_25_, P_75_)		0.9 (0.8, 1.1)	1.0 (0.8, 1.1)	0.19
Serum cystatin C, mg/L, median (P_25_, P_75_)		0.93 (0.82,1.10)	0.84 (0.76, 1.10)	0.0002
Measured glomerular filtration rate, ml/min/1.73 m^2^, median (P_25_, P_75_)		101 (85, 116)	109 (95, 125)	0.0034
eGFR_cr_, ml/min/1.73 m^2^, median (P_25_, P_75_)		103 (85, 118)	103 (92, 114)	0.84
eGFR_cys_, ml/min/1.73 m^2^, median (P_25_, P_75_)		87 (70,103)	101 (81, 112)	0.0001
eGFR_cr-cys_, ml/min/1.73 m^2^, median (P_25_, P_75_)		95 (81, 109)	100 (89, 114)	0.012
Taking antiretroviral therapy, n (%)		171 (91)	-	-
Taking tenofovir, n (%)		127 (68)	-	-
Nadir CD4 count, cells/mm^3^, median (P_25_, P_75_)		145 (42, 301)	-	-
Current CD4 count, cells/mm^3^, median (P_25_, P_75_)		464 (248, 627)	-	-
HIV RNA >400 copies/mL, n (%)		38 (20)	-	-
HIV RNA in subjects with values >400 copies/mL, median (P_25_, P_75_)		11,680 (4,562, 62,084)	-	-

P_25_ and P_75_, 25^th^ and 75^th^ percentiles, respectively; eGFR_cr_, eGFR_cys_, and eGFR_cr-cys_ are glomerular filtration rates estimated by CKD-EPI equations using plasma creatinine, cystatin C, and both biomarkers, respectively.

a Activated CD4 or CD8 T-cells defined as expressing both CD38 and HLA-DR surface markers.

**Table 2:** Performance of glomerular filtration rate estimating equations in HIV-positive and HIV-negative participants.

Performance measure		HIV-positive	HIV-negative	P value^a^
Accuracy^b^ (95% CI)	eGFR_cr_	89 (83, 93)	96 (90, 99)	0.048
	eGFR_cys_	79 (72, 85)	88 (80, 94)	0.075
	eGFR_cr-cys_	91 (85, 94)	97 (91, 99)	0.055
P value^c^	eGFR_cr_ vs. eGFR_cys_	**0.0044**	0.046	-
	eGFR_cr_ vs. eGFR_cr-cys_	0.51	0.71	-
	eGFR_cys_ vs eGFR_cr-cys_	**<0.0001**	**0.0027**	-
Bias^d^ (P_25_, P_75_)	eGFR_cr_	−1.1 (−12.4, 10.4)	−8.8 (−18.0, 2.9)	**0.0013**
	eGFR_cys_	−16.3 (−27.8, −1.8)	−12.9 (−23.8, 0.8)	0.19
	eGFR_cr-cys_	−7.2 (−19.8, 2.2)	−9.7 (−19.1, 0.7)	0.56
P value^c^	eGFR_cr_ vs. eGFR_cys_	**<0.0001**	0.014	-
	eGFR_cr_ vs. eGFR_cr-cys_	**<0.0001**	0.23	-
	eGFR_cys_ vs eGFR_cr-cys_	**<0.0001**	**0.0002**	-
Precision^e^ (95% CI)	eGFR_cr_	22.8 (18.4, 27.3)	20.9 (15.1, 26.7)	0.50
	eGFR_cys_	25.9 (22.1, 29.7)	24.5 (18.9, 30.2)	0.61
	eGFR_cr-cys_	22.0 (18.1, 25.9)	19.8 (13.2, 26.4)	0.49
P value^c^	eGFR_cr_ vs. eGFR_cys_	0.43	0.65	-
	eGFR_cr_ vs. eGFR_cr-cys_	0.43	0.27	-
	eGFR_cys_ vs eGFR_cr-cys_	0.12	0.10	-

CI, confidence interval; eGFR_cr_, eGFR_cys_, and eGFR_cr-cys_ are glomerular filtration rates estimated by CKD-EPI equations using plasma creatinine, cystatin C, and both biomarkers, respectively; P_25_ and P_75_, 25^th^ and 75^th^ percentiles, respectively.

a Comparisons of a single equation between the HIV-positive and HIV-negative groups. P values in bold font indicate difference is statistically significant accounting for multiple comparisons (see text).

b Accuracy defined as percentage of estimated GFR values within 30% of measured GFR.

c Comparisons of a different equations within the HIV-positive or HIV-negative group. P values in bold font indicate difference is statistically significant accounting for multiple comparisons (see text).

d Bias defined as difference between estimated GFR and measured GFR (mL/min/1.73 m^2^).

e Precision defined as interquartile range of bias.

**Table 3:** Factors associated with glomerular filtration rate estimating equation accuracy^a^ in HIV-positive and HIV-negative participants.

Factor		HIV-positive		HIV-negative	
		eGFR_cr_	eGFR_cys_	eGFR_cr_	eGFR_cys_
Age, years	≤49	87 (79, 93)	80 (70, 87)	92 (81, 98)	94 (84, 99)
	>49	90 (82, 95)	79 (69, 87)	100 (92, 100)	81 (67, 91)
	P value^b^	0.64	1.00	0.12	0.064
Body mass index, kg/m^2^	≤26	88 (79, 93)	77 (67, 85)	96 (85, 99)	84 (70, 93)
	>26	90 (82, 95)	82 (72, 89)	96 (87, 99)	91 (79, 97)
	P value^b^	0.65	0.47	1.00	0.38
Sex	Female	83 (72, 91)	78 (66, 87)	89 (65, 99)	100 (81, 100)
	Male	92 (85, 96)	80 (71, 86)	97 (91, 100)	85 (75, 92)
	P value^b^	0.093	0.85	0.15	0.12
mGFR, mL/min/1.73 m^2^	<90	80 (68, 89)	70 (57, 82)	94 (71, 99)	88 (64, 99)
	≥90	93 (87, 97)	83 (75, 89)	96 (90, 99)	88 (78, 94)
	P value^b^	0.014	0.051	0.54	1.00
Hepatitis C serostatus	Negative	88 (80, 94)	85 (76, 92)	94 (86, 98)	91 (82, 97)
	Positive	89 (81, 94)	73 (64, 82)	100 (88, 100)	79 (59, 92)
	P value^b^	1.00	0.066	0.32	0.10
High-sensitivity C-reactive protein, mg/dL	≤1.8	91 (83, 96)	79 (70, 87)	98 (89, 100)	82 (68, 91)
	>1.8	88 (79, 94)	80 (70, 88)	94 (83, 99)	94 (83, 99)
	P value^b^	0.64	0.86	0.62	0.12
Percentage activated CD4 cells	≤Median^c^	90 (82, 95)	92 (84, 97)	92 (80, 98)	90 (78, 97)
	>Median^c^	88 (79, 94)	69 (58, 78)	100 (93,100)	86 (73, 94)
	P value^b^	0.81	**0.0001**	0.12	0.76
Percentage activated CD8 cells	≤Median^d^	91 (83, 96)	92 (84, 97)	94 (83, 99)	87 (75, 95)
	>Median^d^	87 (78, 93)	69 (58, 78)	98 (89, 100)	88 (76, 95)
	P value^b^	0.48	**0.0001**	0.36	1.00
Taking antiretroviral therapy	Yes	89 (84, 94)	82 (75, 87)		
	No	81 (54, 96)	50 (25, 75)		
	P value^b^	0.40	**0.0064**		
Nadir CD4, cells/mm^3^	>150	86 (77, 92)	80 (71, 88)		
	≤150	92 (84, 96)	78 (68, 86)		
	P value^b^	0.25	0.72		
Current CD4, cells/mm^3^	>450	89 (81, 94)	88 (80, 94)		
	≤450	89 (80, 94)	70 (59, 79)		
	P value^b^	1.00	**0.0032**		
HIV RNA, copies/ml	≤400	91 (85, 95)	87 (80, 92)		
	>400	79 (63, 90)	50 (33, 67)		
	P value^b^	0.043	**<0.0001**		

eGFR_cr_ and eGFR_cys_ are glomerular filtration rates estimated by CKD-EPI equations using plasma creatinine and cystatin C, respectively; mGFR, measured glomerular filtration rate by iohexol clearance.

a Accuracy shown as percent of estimated GFR values within 30% of measured GFR values (95% confidence interval).

b P values in bold font indicate difference is statistically significant accounting for multiple comparisons (see text).

c Medians 8.3% and 3.8% in HIV-positive and HIV-negative groups, respectively.

d Medians 30.7% and 10.7% in HIV-positive and HIV-negative groups, respectively.

**Table 4:** Factors associated with glomerular filtration rate equation bias^a^ in HIV-positive and HIV-negative participants.

Factor		HIV-positive		HIV-negative	
		eGFR_cr_	eGFR_cys_	eGFR_cr_	eGFR_cys_
Age, years	≤49	−1.9 (−16.2, 10.7)	−16.4 (−24.9, 1.3)	−9.9 (−23.2, 3.8)	−12.6 (−21.6, 2.0)
	>49	0.7 (−7.9, 9.5)	−14.2 (−29.2, 2.0)	−5.8 (−17.6, 1.6)	−13.9 (−25.9, 0.7)
	P value^b^	0.30	0.76	0.50	0.44
Body mass index, kg/m^2^	≤26	−2.6 (−12.6, 10.4)	−17.7 (−30.7, −1.8)	−8.9 (−23.0, 1.4)	−13.0 (−21.6, 0.1)
	>26	0.9 (−11.3, 10.3)	−12.3 (−24.3, −1.6)	−8.0 (−15.6, 3.8)	−12.6 (−25.9, 1.9)
	P value^b^	0.61	0.33	0.18	0.84
Sex	Female	6.5 (−5.3, 17.5)	−12.8 (−26.2, −1.6)	−1.4 (−8.0, 13.2)	−13.3 (−17.8, −4.5)
	Male	−4.6 (−14.5, 7.0)	−17.1 (−28.8, −1.9)	−10.3 (−20.4, −1.4)	−12.3 (−24.5, 3.1)
	P value^b^	**0.0011**	0.66	**0.0049**	0.78
mGFR, mL/min/1.73 m^2^	<90	4.5 (−5.0, 18.7)	−7.9 (−21.7, 0.4)	6.0 (−5.2, 16.3)	−7.5 (−13.9, 8.0)
	≥90	−4.5 (−16.2, 7.5)	−19.9 (−30.9, −3.4)	−10.3 (−20.6, −1.0)	−13.5 (−26.0, −2.5)
	P value^b^	**0.0001**	**0.0039**	**0.0002**	**0.0080**
Hepatitis C serostatus	Negative	−0.9 (−12.4, 13.8)	−6.8 (−24.2, 2.1)	−9.9 (−19.6, 4.2)	−8.0 (−20.3, 5.0)
	Positive	−1.7 (−11.7, 9.1)	−21.1 (−28.8, −6.0)	−8.8 (−15.9, 1.2)	−21.6 (−33.4, −12.9)
	P value^b^	0.67	**0.0012**	0.97	**0.0006**
High-sensitivity C-reactive protein, mg/dl	≤1.8	−1.4 (−10.3, 10.3)	−16.3 (−30.2, 2.0)	−9.7 (−20.2, 1.6)	−13.5 (−25.0, 1.9)
	>1.8	−0.2 (−12.6, 10.7)	−15.9 (−25.0, 1.1)	−7.0 (−15.8, 3.8)	−10.4 (−22.4, −3.3)
	P value^b^	0.96	0.72	0.48	0.79
Percentage activated CD4 cells	≤Median^c^	−2.0 (−13.5, 8.0)	−6.4 (−22.6, 0.3)	−8.8 (−18.9, 5.5)	−11.1 (−18.3, 1.9)
	>Median^c^	−1.1 (−11.0, 13.0)	−21.6 (−33.6, −8.4)	−8.9 (−16.7, 0.6)	−16.3 (−25.2, 2.5)
	P value^b^	0.53	**<0.0001**	0.87	0.12
Percentage activated CD8 cells	≤Median^c^	−3.9 (−14.5, 9.2)	−7.6 (−21.7, 0.7)	−6.1 (−19.0, 5.3)	−11.4 (−22.6, 4.5)
	>Median^c^	1.0 (−9.7, 12.8)	−21.7 (−31.9, −5.9)	−10.0 (−17.6, −1.2)	−13.0 (−25.2, 3.3)
	P value^b^	0.20	**0.0003**	0.34	0.25
Taking antiretroviral therapy	Yes	−1.0 (−12.4, 10.7)	−14.2 (−27.4, −1.1)		
	No	−3.0 (−16.5, 8.3)	−25.7 (−47.0, −21.0)		
	P value^b^	0.61	**0.0014**		
Nadir CD4 count, cells/mm^3^	>150	−5.3 (−19.8, 7.0)	−20.3 (−30.7, −3.3)		
	≤150	4.0 (−7.2, 12.9)	−11.5 (−25.0, 0)		
	P value^b^	**0.0009**	0.056		
CD4 count, cells/mm^3^	>450	−2.0 (−14.5, 8.0)	−12.2 (−24.2, −0.4)		
	≤450	−0.1 (−9.5, 13.6)	−19.8 (−31.6, −4.8)		
	P value^b^	0.20	0.032		
HIV RNA, copies/ml	≤400	−0.2 (−12.0, 9.5)	−10.0 (−24.1, −0.6)		
	>400	−3.6 (−12.4, 12.9)	−28.0 (−45.4, −18.7)		
	P value^b^	0.63	**<0.0001**		

eGFR_cr_ and eGFR_cys_ are glomerular filtration rates estimated by CKD-EPI equations using plasma creatinine and cystatin C, respectively; mGFR, measured glomerular filtration rate by iohexol clearance.

a Bias defined as median difference between estimated glomerular filtration rate (GFR) and measured GFR (25^th^ percentile, 75^th^ percentile).

b P values in bold font indicate difference is statistically significant accounting for multiple comparisons (see text).

c Medians 8.3% and 3.8% in HIV-positive and HIV-negative groups, respectively.

d Medians 30.7% and 10.7% in HIV-positive and HIV-negative groups, respectively.

As a supplemental analysis, we evaluated HIV-related factors associated with eGFR_cys_ accuracy in HIV-positive subjects in a multivariate logistic regression model. Factors significantly associated with eGFR_cys_ accuracy in univariate analysis were selected for inclusion in the multivariate model by stepwise backward selection, where factors with P>0.05 were removed from the model, with the covariate for use of antiretroviral therapy forced into the final model. Additionally, we explored the association between activated CD8 cells and bias for eGFR_cr_ and eGFR_cys_ using scatterplots, Spearman correlation coefficients, and linear regression. We used REDCap for data collection and management [17] and Stata version 10 (Stata Corp, College Station, TX) for the statistical analysis.

## Results

Table 1 summarizes the clinical characteristics of the study participants. HIV-positive and HIV-negative groups were similar in age, BMI, race, and smoking status. The HIV-positive group had higher proportions of women and hepatitis C seropositive subjects. HIV-positive subjects were more likely HIV-negative subjects to have a history of hypertension and cardiovascular disease, although systolic blood pressure and glycosylated hemoglobin levels were higher in HIV-negative subjects. There was no significant difference in hsCRP between the two groups, although levels of activated CD4 and CD8 cells were substantially higher in the HIV-positive participants. The median urine albumin-creatinine ratio was similar in the two groups, although HIV-positive subjects were significantly more likely to have an albumin-creatinine ratio >30 mg/g. mGFR was significantly lower in HIV-positive than HIV-negative subjects. Creatinine and eGFR_cr_ were similar in the two groups. In contrast, cystatin C was significantly higher and eGFR_cys_ and eGFR_cr-cys_ were significantly lower in HIV-positive subjects.

Most HIV-positive subjects were taking antiretroviral therapy and had HIV RNA <400 copies/mL. Sixty eight percent of HIV-positive subjects were taking tenofovir, but no participant was taking a cobicistat-containing preparation. CD4 and CD8 activation in HIV- positive participants was strongly associated with HIV RNA suppression, as has been reported previously [18], [19]. In HIV-positive participants with HIV RNA >400 copies/mL the median value for activated CD4 T-cells was 18% compared with 7% in those with HIV RNA ≤400 copies/mL (P<0.0001). Similarly, the median values of activated CD8 T-cells were 54% and 27% in HIV-positive subjects with HIV RNA >400 or ≤400 copies/mL, respectively (P<0.0001).

**Figure 1:**
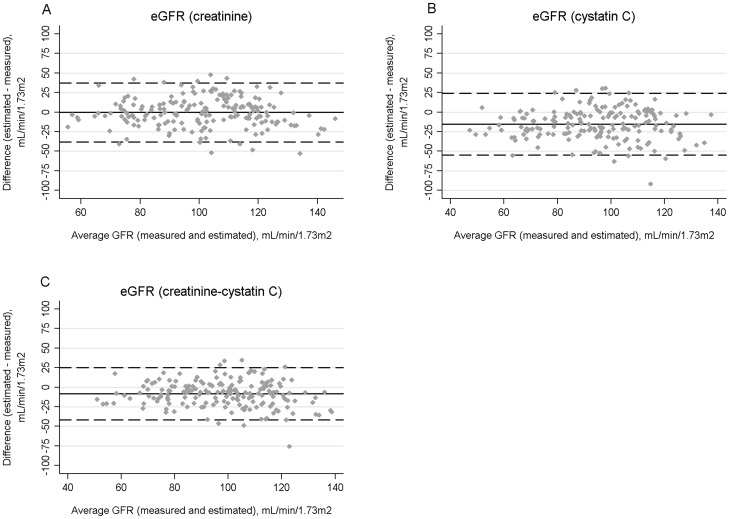
Bland-Altman plots for estimated and measured glomerular filtration rate (GFR) in HIV-positive participants using the CKD-EPI equations for serum creatinine (A), cystatin C (B), or both biomarkers (C). The average GFR (measured and estimated) is shown on the X axes. Bias, defined as the difference between estimated and measured GFR, is displayed on the Y axes. The average biases are represented by the horizontal solid lines and the horizontal dashed lines represent 2 standard deviations above and below the averages.

**Figure 2:**
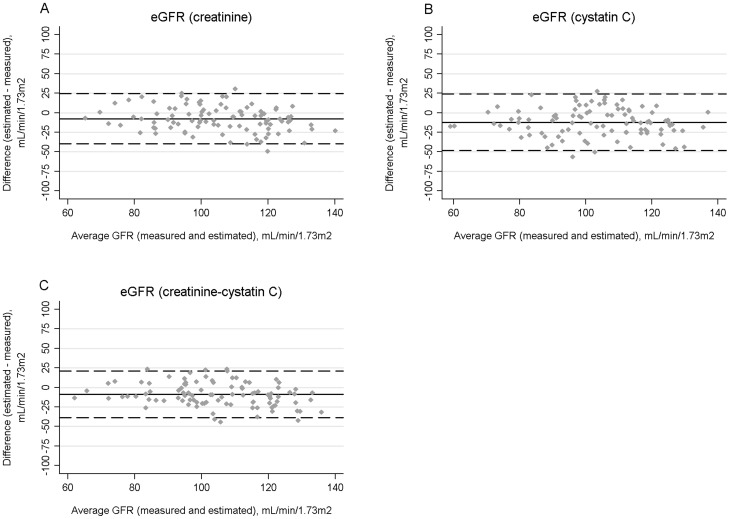
Bland-Altman plots for estimated and measured glomerular filtration rate (GFR) in HIV-negative participants using the CKD-EPI equations for serum creatinine (A), cystatin C (B), or both biomarkers (C). The average GFR (measured and estimated) is shown on the X axes. Bias, defined as the difference between estimated and measured GFR, is displayed on the Y axes. The average biases are represented by the horizontal solid lines and the horizontal dashed lines represent 2 standard deviations above and below the averages.

Table 2 shows performance measures of the three estimating equations. All three equations tended to be less accurate in HIV-positive than HIV-negative subjects, although differences were not statistically significant when accounting for multiple comparisons. Additionally, all three equations tended to underestimate mGFR. The eGFR_cr_ equation was significantly less biased in HIV-positive than HIV-negative subjects. The precision of the three equations was similar in both groups. Bland-Altman plots of the three equations in HIV-positive and HIV-negative subjects are shown in Figures 1 and 2.

Comparing equations with one another in the subgroups, eGFR_cys_ was significantly less accurate within 30% of mGFR than both eGFR_cr_ and eGFR_cr-cys_ in the HIV-positive group (accuracy of 79% compared to 89% and 91%, respectively), and eGFR_cys_ was significantly less accurate than eGFR_cr-cys_ in the HIV-negative group. Assessment of accuracy within 10% of mGFR showed a similar pattern. Specifically, among HIV-positive subjects accuracies within 10% were 31%, 45%, and 42% for eGFR_cys_, eGFR_cr_, and eGFR_cr-cys_. Among HIV-negative subjects, accuracies within 10% were 31%, 41%, and 43% for eGFR_cys_, eGFR_cr_, and eGFR_cr-cys_, respectively.

In the HIV-positive group eGFR_cys_ was significantly more biased than both eGFR_cr_ and eGFR_cr-cys_ (median bias of −16.3 compared to −1.1 and −7.2 mL/min/1.73 m^2^, respectively), and eGFR_cr-cys_ was significantly more biased than eGFR_cr_. In the HIV-negative group, eGFR_cys_ was significantly more biased than eGFR_cr-cys_. eGFR_cys_ tended to be the least precise of the estimating equations however, there were no statistically significant differences between equations in either group.

Table 3 shows the associations of clinical factors with the accuracy of eGFR_cr_ and eGFR_cys_ in the HIV-positive and HIV-negative groups. In the HIV-positive group, lower eGFR_cys_ accuracy was significantly associated with activated CD4 and CD8 percentages above the medians, not taking antiretroviral therapy, current CD4 count <450 cells/mm^3^, and HIV RNA >400 copies/mL. eGFR_cys_ accuracy was not significantly associated with age, BMI, sex, mGFR strata, hepatitis C serostatus, hsCRP, or nadir CD4 count. No factors were significantly associated with the accuracy of eGFR_cr_ in either group and no factors were significantly associated with eGFR_cys_ accuracy in the HIV-negative group, accounting for multiple comparisons.

Table 4 shows the associations of clinical factors with eGFR_cr_ and eGFR_cys_ bias. Sex was significantly associated with eGFR_cr_ bias in both groups. mGFR stratum was significantly associated with eGFR_cr_ and eGFR_cys_ bias in both groups, with larger underestimation at mGFR ≥90 versus <90 mL/min/1.73 m^2^. Hepatitis C serostatus was associated with eGFR_cys_ bias in both the HIV-positive and HIV-negative groups, with larger underestimation in hepatitis C seropositive than seronegative subjects. In the HIV-positive group, eGFR_cys_ bias was significantly associated with not taking antiretroviral therapy, activated CD4 and CD8 percentages above the medians, and HIV RNA >400 copies/mL. Finally, nadir CD4 count was significantly associated with eGFR_cr_ bias, with modest underestimation and overestimation at nadir CD4 counts >150 and ≤150 cells/mm^3^, respectively. Age, BMI, hsCRP, and current CD4 cell count were not significantly associated with eGFR_cr_ or eGFR_cys_ bias in either group, accounting for multiple comparisons.

In a supplemental analysis, we assessed factors associated with eGFR_cys_ accuracy in a multivariate logistic regression model, in which the covariate for antiretroviral therapy use was forced into the model and other candidate variables (HIV RNA >400 copies/mL, current CD4 count, activated CD8 cells, and activated CD4 cells) were selected by backward stepwise selection. Current CD4 count and activated CD4 were dropped from the model. Use of antiretroviral therapy, which was significantly associated with an increased odds of eGFR_cys_ accuracy in unadjusted analysis (odds ratio [OR], 4.5; 95% confidence interval [CI] 1.6, 13.0), was not significantly associated with accuracy in the multivariate model (OR, 1.2; 95% CI 0.3, 4.2). Both HIV RNA >400 copies/mL (OR, 0.2; 95% CI 0.1, 0.7) and activated CD8 percentage above the median (OR, 0.3, 95% CI 0.1, 0.9) were significantly associated with lower likelihood of eGFR_cys_ accuracy in the multivariate model.

**Figure 3:**
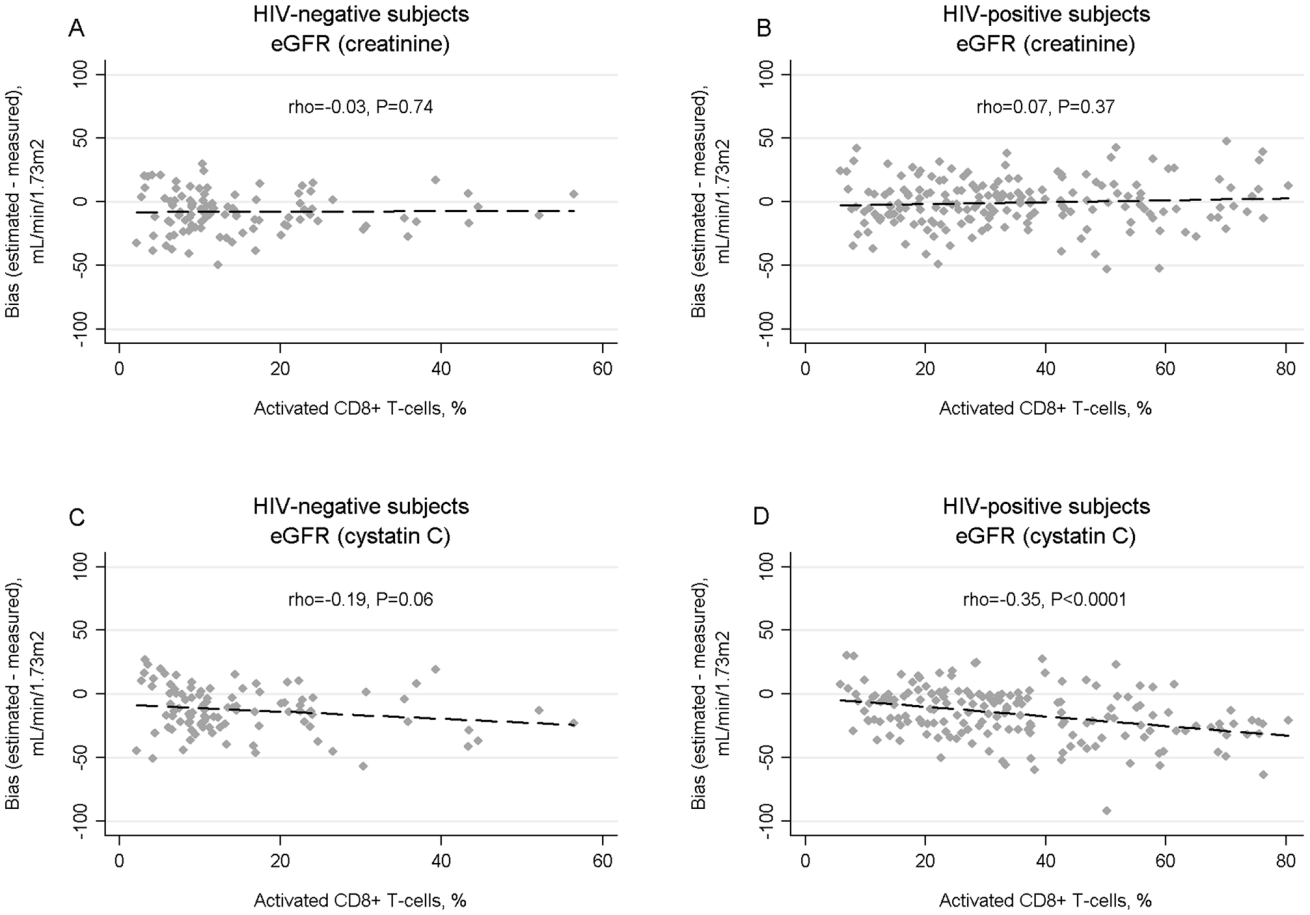
Correlation of estimated glomerular filtration rate (eGFR) bias, defined as the difference between eGFR and measured GFR, with percentage of activated CD8 T cells (CD38+ and HLA-DR+) using the creatine-based CKD-EPI equation in HIV-negative (A) and HIV-positive (B) subjects, and the cystatin C-based CKD-EPI equation in HIV negative (C) and HIV-positive (D) subjects. The percentage of CD8+ T cells with an activated phenotype is shown on the X axes (note, different scales for HIV-positive and HIV-negative groups). Rho is the spearman rank correlation coefficient, which may vary between −1 and 1. The dashed lines represent least-squares regression lines.

Figure 3 shows the relationships between activated CD8 T-cells and the bias of eGFR_cr_ and eGFR_cys_ in HIV-positive and HIV-negative participants. The percentage activated CD8 T-cells was not significantly correlated with eGFR_cr_ bias in either group. In contrast, activated CD8 T-cells were significantly negatively correlated with eGFR_cys_ bias in HIV-positive (rho = −0.35, P<0.0001) but not HIV-negative subjects. In HIV-positive subjects, each 20% increase in activated CD8 cells was associated a significant bias difference of −7.5 mL/min/1.73 m^2^ (95% CI, −10.6, −4.5) for eGFR_cys_, but no significant bias difference for eGFR_cr_ (1.5 mL/min/1.73 m^2^; 95% CI −1.5, 4.5). In HIV-negative subjects, each 20% increase in activated CD8 cells was associated a borderline significant bias difference of −5.7 mL/min/1.73 m^2^ (95% CI, −12.2, 0.6) for eGFR_cys_, but no bias difference for eGFR_cr_ (0.4 mL/min/1.73 m^2^; 95% CI −5.4, 6.2).

## Discussion

Our first objective in this analysis was to compare the performance of CKD-EPI GFR equations in HIV-positive and HIV-negative participants. All equations tended to be less accurate in the HIV-positive group, although no differences were statistically significant when accounting for multiple comparisons. Our second objective was to compare the performance of equations with one another, with a focus on the HIV-positive group. We found that eGFR_cys_ was significantly less accurate than eGFR_cr_ or eGFR_cr-cys_ in HIV-positive subjects, and that eGFR_cys_ underestimated mGFR by a median of 16.3 ml/min/1.73 m^2^ in HIV-positive subjects, a significantly larger bias than either eGFR_cr_ or eGFR_cr-cys_.

Our third objective was to assess factors associated with the accuracy and bias of eGFR_cr_ and eGFR_cys_, in order to identify factors that might differentially affect the performance of these intrinsic GFR biomarkers. Markers of uncontrolled HIV-infection (non-use of antiretroviral therapy, lower current CD4 cell count, HIV RNA >400 copies/mL, and higher T-cell activation) were strongly associated with lower accuracy and larger bias of eGFR_cys_. In a multivariate model, the association between use of antiretroviral therapy and eGFR_cys_ accuracy appeared to be completely explained by HIV RNA suppression and percentage of activated CD8 cells. Additionally, we found that hepatitis C seropositivity was associated with a larger eGFR_cys_ bias in both HIV-positive and HIV-negative subjects. In contrast, none of these HIV-related factors was significantly associated with eGFR_cr_ performance.

Our results should be considered in context of other studies that have examined the performance of GFR estimating equations in HIV-positive individuals. First, we found that all three CKD-EPI equations tended to be less accurate in HIV- positive than in HIV-negative subjects. To date there are few published data directly comparing equation performance in HIV-positive and HIV-negative subjects, although two studies [6], [7] noted lower CKD-EPI equation accuracy in HIV-positive subjects compared to historical data from the general population [15].

Second, among HIV-positive subjects, we found that the eGFR_cr_ and eGFR_cr-cys_ equations had similar accuracy and were both significantly more accurate than eGFR_cys_. Additionally, eGFR_cr_ was significantly less biased than both eGFR_cys_ and eGFR_cr-cys_. A study of 203 HIV-positive European subjects, in with mGFR measurements by iohexol clearance, found that eGFR_cys_ accuracy was slightly lower than eGFR_cr_ or eGFR_cr-cys_, although differences were not statistically significant, and bias was similar in the three equations. In contrast, Inker and colleagues evaluated CKD-EPI equations in 200 HIV-positive participants [6] and found that eGFR_cr_ and eGFR_cys_ had similar accuracy and bias, while eGFR_cr-cys_ was significantly more accurate than eGFR_cys_ and non-significantly more accurate than eGFR_cr_. Two other studies that used earlier cystatin C equations variably found cystatin C-based GFR to be more accurate [20] and less accurate [21] than creatinine-based equations. In aggregate, these studies provide little support for the use of cystatin C to improve GFR estimation in HIV-positive individuals, particularly when used alone. Our study and that of Inker and colleagues found eGFR_cr-cys_ to have the highest accuracy, although the differences between eGFR_cr-cys_ and eGFR_cr_ were small and not statistically significant in either study.

Third, a substantive finding in our study was that accuracy and bias of eGFR_cys_ were strongly associated with HIV-related factors, notably HIV RNA suppression, while eGFR_cr_ performance had no association with these factors. Prior studies have described correlations between cystatin C and antiretroviral therapy, HIV RNA, and CD4 counts [22]–[25]. For example, in the Strategies for Management of Antiretroviral Therapy (SMART) study, subjects randomized to discontinue or defer antiretroviral therapy were significantly more likely to have an increase in serum cystatin C from baseline than those randomized to continuous therapy, while creatinine changes were not significantly different in the two arms [24]. However, it has been uncertain if cystatin C was reflecting true reductions in GFR, or if cystatin C was confounded by HIV-related non-GFR factors. Our results support the latter hypothesis. A recent European study also observed a trend for higher accuracy with eGFR_cr_ as compared to eGFR_cys_ in subjects with a detectable HIV RNA, lending support to our findings [7]. However, Inker and colleagues reported no significant differences in eGFR_cys_ accuracy or bias according to HIV RNA strata, although this study had only 14 HIV-positive subjects with HIV RNA >1,000 copies/mL, limiting their ability to detect a difference in the subset without viral suppression [6].

A novel finding in our study was the strong association between markers of T-cell activation and eGFR_cys_ accuracy and bias. Compared to HIV-negative persons, HIV-positive individuals have a higher prevailing state of immune activation - as measured by the percentages of activated CD4 and CD8 cells - which may be driven by microbial translocation resulting from disruption of the gut-associated lymphoid tissue in HIV infection [13]. T-cell activation markers have long been recognized as predictors of HIV disease progression and mortality, independent of CD4 and viral load [26]. Levels of T-cell activation has been found to decline markedly with antiretroviral therapy and viral suppression, although not to levels seen in HIV-negative persons.

Our results imply a strong positive correlation between levels of activated CD4 and CD8 cells and cystatin C that was independent of GFR. In particular, we found that underestimation bias of eGFR_cys_ increased linearly with increasing percentage of activated CD8 cells in HIV-positive subjects. To our knowledge, no prior studies have assessed associations between T-cell activation markers and cystatin C or eGFR_cys_ performance. The association between cystatin C and inflammation or immune activations in HIV-positive individuals may explain the stronger observed association of cystatin C and mortality than creatinine and mortality in HIV-positive persons [27], [28], and in the general population [29]. In contrast, we found no association between the inflammation marker, hsCRP, and eGFR_cys_ performance. This suggests that the mechanism by which HIV-related T-cell activation affects plasma cystatin C is distinct from inflammatory pathways reflected by hsCRP.

Our results have clinical implications. First, all three CKD-EPI equations tended to be less accurate in HIV-positive than HIV-negative subjects. Although these differences were not statistically significant when accounting for multiple comparisons, they may be clinically relevant and evaluation in larger comparative studies is indicated. Second, among the CKD-EPI equations, there was no evidence that cystatin C, either alone or in combination with creatinine, appreciably improved GFR estimation compared to using creatinine alone. Third, we found that antiretroviral therapy use, detectable HIV RNA, and lower CD4 counts were strongly associated with the accuracy and bias of eGFR_cys_, but not with eGFR_cr_. Moreover, these associations may be mediated by T-cell activation, although additional research is needed to confirm this association. This implies that cystatin C may be a suboptimal GFR index in HIV-positive persons, particularly in those not taking antiretroviral therapy or with uncontrolled viremia.

The strengths of our study include use of iohexol clearance – an exogenous gold-standard GFR measurement method, use of standardized plasma creatinine and cystatin C measurements, and inclusion of a demographically similar HIV-negative comparison group. Our study also has limitations. First, the sample size was relatively small, and we may have insufficient power to detect potentially clinically significant differences. Second, over 90% of our study population was African American, which precluded comparisons by racial groups. Our results may not be generalizable to HIV-positive individuals of other racial backgrounds. Third, our cohort was restricted to individuals with MDRD GFR >60 ml/min/1.73 m^2^ and equation performance could not be assessed in persons with poorer kidney function.

In conclusion, we found that CKD-EPI equations based on creatinine and cystatin C tended to be less accurate in HIV-positive than HIV-negative subjects. eGFR_cys_ was significantly less accurate and more biased than both the widely-used eGFR_cr_ equation and the combined eGFR_cr-cys_ equation in HIV-positive individuals. Moreover, eGFR_cys_ performance was strongly affected by uncontrolled HIV disease and T-cell activation indices. In contrast, eGFR_cr_ performance was not modified by HIV-related factors or T-cell activation.

## References

[1] Dharnidharka VR, Kwon C, Stevens G (2002) Serum cystatin C is superior to serum creatinine as a marker of kidney function: a meta-analysis. Am J Kidney Dis 40: 221–226. 12148093 10.1053/ajkd.2002.34487

[2] Gagneux-Brunon A, Mariat C, Delanaye P (2012) Cystatin C in HIV-infected patients: promising but not yet ready for prime time. Nephrol Dial Transplant 27: 1305–1313. 22399493 10.1093/ndt/gfs001

[3] Luc G, Bard JM, Lesueur C, Arveiler D, Evans A, et al (2006) Plasma cystatin-C and development of coronary heart disease: The PRIME Study. Atherosclerosis 185: 375–380. 16046222 10.1016/j.atherosclerosis.2005.06.017

[4] Earley A, Miskulin D, Lamb EJ, Levey AS, Uhlig K (2012) Estimating equations for glomerular filtration rate in the era of creatinine standardization: a systematic review. Ann Intern Med 156: 785–W-278, 785-795, W-270, W-271, W-272, W-273, W-274, W-275, W-276, W-277, W-278. 22312131 10.7326/0003-4819-156-11-201203200-00391

[5] Levey AS, Stevens LA, Schmid CH, Zhang YL, Castro AF 3rd, et al (2009) A new equation to estimate glomerular filtration rate. Ann Intern Med 150: 604–612. 19414839 10.7326/0003-4819-150-9-200905050-00006 PMC2763564

[6] Inker LA, Wyatt C, Creamer R, Hellinger J, Hotta M, et al (2012) Performance of Creatinine and Cystatin C GFR Estimating Equations in an HIV-Positive Population on Antiretrovirals. J Acquir Immune Defic Syndr 61: 302–309. 22842844 10.1097/QAI.0b013e31826a6c4f PMC3598619

[7] Gagneux-Brunon A, Delanaye P, Maillard N, Fresard A, Basset T, et al (2013) Performance of creatinine and cystatin C-based glomerular filtration rate estimating equations in a European HIV-positive cohort. AIDS 10.1097/QAD.0b013e32835fac30 23435293

[8] Vlahov D, Anthony JC, Munoz A, Margolick J, Nelson KE, et al (1991) The ALIVE study, a longitudinal study of HIV-1 infection in intravenous drug users: description of methods and characteristics of participants. NIDA Res Monogr 109: 75–100. 1661376

[9] Levey AS, Bosch JP, Lewis JB, Greene T, Rogers N, et al (1999) A more accurate method to estimate glomerular filtration rate from serum creatinine: a new prediction equation. Modification of Diet in Renal Disease Study Group. Ann Intern Med 130: 461–470. 10075613 10.7326/0003-4819-130-6-199903160-00002

[10] Myers GL, Miller WG, Coresh J, Fleming J, Greenberg N, et al (2006) Recommendations for improving serum creatinine measurement: a report from the Laboratory Working Group of the National Kidney Disease Education Program. Clin Chem 52: 5–18. 16332993 10.1373/clinchem.2005.0525144

[11] Grubb A, Blirup-Jensen S, Lindstrom V, Schmidt C, Althaus H, et al (2010) First certified reference material for cystatin C in human serum ERM-DA471/IFCC. Clin Chem Lab Med 48: 1619–1621. 21034257 10.1515/CCLM.2010.318

[12] Giorgi JV, Ho HN, Hirji K, Chou CC, Hultin LE, et al (1994) CD8+ lymphocyte activation at human immunodeficiency virus type 1 seroconversion: development of HLA-DR+ CD38− CD8+ cells is associated with subsequent stable CD4+ cell levels. The Multicenter AIDS Cohort Study Group. J Infect Dis 170: 775–781. 7930717 10.1093/infdis/170.4.775

[13] Brenchley JM, Price DA, Schacker TW, Asher TE, Silvestri G, et al (2006) Microbial translocation is a cause of systemic immune activation in chronic HIV infection. Nat Med 12: 1365–1371. 17115046 10.1038/nm1511

[14] Schwartz GJ, Furth S, Cole SR, Warady B, Munoz A (2006) Glomerular filtration rate via plasma iohexol disappearance: pilot study for chronic kidney disease in children. Kidney Int 69: 2070–2077. 16612328 10.1038/sj.ki.5000385

[15] Inker LA, Schmid CH, Tighiouart H, Eckfeldt JH, Feldman HI, et al (2012) Estimating glomerular filtration rate from serum creatinine and cystatin C. N Engl J Med 367: 20–29. 22762315 10.1056/NEJMoa1114248 PMC4398023

[16] Benjamini Y, Hochberg Y (1995) Controlling the False Discovery Rate - a Practical and Powerful Approach to Multiple Testing. Journal of the Royal Statistical Society Series B-Methodological 57: 289–300.

[17] Harris PA, Taylor R, Thielke R, Payne J, Gonzalez N, et al (2009) Research electronic data capture (REDCap)–a metadata-driven methodology and workflow process for providing translational research informatics support. J Biomed Inform 42: 377–381. 18929686 10.1016/j.jbi.2008.08.010 PMC2700030

[18] Benito JM, Lopez M, Lozano S, Martinez P, Gonzalez-Lahoz J, et al (2004) CD38 expression on CD8 T lymphocytes as a marker of residual virus replication in chronically HIV-infected patients receiving antiretroviral therapy. AIDS Res Hum Retroviruses 20: 227–233. 15018711 10.1089/088922204773004950

[19] Beran O, Holub M, Spala J, Kalanin J, Stankova M (2003) Cd38 expression on Cd8+ T cells in Human immunodeficiency virus 1-positive adults treated with HAART. Acta Virol 47: 121–124. 14524479

[20] Praditpornsilpa K, Avihingsanon A, Chaiwatanarat T, Chaiyahong P, Wongsabut J, et al (2012) Comparisons between validated estimated glomerular filtration rate equations and isotopic glomerular filtration rate in HIV patients. AIDS 26: 1781–1788. 22713478 10.1097/QAD.0b013e328356480d PMC3782632

[21] Barraclough K, Er L, Ng F, Harris M, Montaner J, et al (2009) A comparison of the predictive performance of different methods of kidney function estimation in a well-characterized HIV-infected population. Nephron Clin Pract 111: c39–48. 19052469 10.1159/000178978

[22] Estrella MM, Parekh RS, Astor BC, Bolan R, Evans RW, et al (2011) Chronic kidney disease and estimates of kidney function in HIV infection: a cross-sectional study in the multicenter AIDS cohort study. J Acquir Immune Defic Syndr 57: 380–386. 21646913 10.1097/QAI.0b013e318222f461 PMC3159728

[23] Mauss S, Berger F, Kuschak D, Henke J, Hegener P, et al (2008) Cystatin C as a marker of renal function is affected by HIV replication leading to an underestimation of kidney function in HIV patients. Antivir Ther 13: 1091–1095. 19195336

[24] Mocroft A, Wyatt C, Szczech L, Neuhaus J, El-Sadr W, et al (2009) Interruption of antiretroviral therapy is associated with increased plasma cystatin C. AIDS 23: 71–82. 19050388 10.1097/QAD.0b013e32831cc129 PMC2761385

[25] Jones CY, Jones CA, Wilson IB, Knox TA, Levey AS, et al (2008) Cystatin C and creatinine in an HIV cohort: the nutrition for healthy living study. Am J Kidney Dis 51: 914–924. 18455851 10.1053/j.ajkd.2008.01.027 PMC4430838

[26] Giorgi JV, Hultin LE, McKeating JA, Johnson TD, Owens B, et al (1999) Shorter survival in advanced human immunodeficiency virus type 1 infection is more closely associated with T lymphocyte activation than with plasma virus burden or virus chemokine coreceptor usage. J Infect Dis 179: 859–870. 10068581 10.1086/314660

[27] Choi A, Scherzer R, Bacchetti P, Tien PC, Saag MS, et al (2010) Cystatin C, albuminuria, and 5-year all-cause mortality in HIV-infected persons. Am J Kidney Dis 56: 872–882. 20709438 10.1053/j.ajkd.2010.05.019 PMC3164880

[28] Driver TH, Scherzer R, Peralta CA, Tien PC, Estrella MM, et al (2013) Comparisons of creatinine and cystatin C for detection of kidney disease and prediction of all-cause mortality in HIV-infected women. AIDS 27: 2291–2299. 23669156 10.1097/QAD.0b013e328362e874 PMC3919542

[29] Peralta CA, Shlipak MG, Judd S, Cushman M, McClellan W, et al (2011) Detection of chronic kidney disease with creatinine, cystatin C, and urine albumin-to-creatinine ratio and association with progression to end-stage renal disease and mortality. JAMA 305: 1545–1552. 21482744 10.1001/jama.2011.468 PMC3697771

