# Correction: HIV Viremia and T-cell Activation Differentially Affect the Performance of Glomerular Filtration Rate Equations Based on Creatinine and Cystatin C

**DOI:** 10.1371/journal.pone.0215630

**Published:** 2019-04-16

**Authors:** Bhavna Bhasin, Bryan Lau, Mohamed G. Atta, Derek M. Fine, Michelle M. Estrella, George J. Schwartz, Gregory M. Lucas

After publication of this article [1], it came to light that there were errors in the reported glomerular filtration rate (GFR) estimates.

The two-fold purpose of this paper [1] was to 1) compare accuracy and bias of widely used glomerular filtration rate (GFR) estimating equations to a gold-standard GFR measure (iohexol disappearance from plasma) in HIV-positive and HIV-negative volunteers, and 2) to assess factors associated with bias and accuracy of the creatinine-based and cystatin C-based equations. Recently, our co-investigators, who performed the laboratory analyses and calculations for the iohexol GFR, identified a drift that occurred in their measurement of iohexol (prior to this study) that led to an across-the-board underestimation of iohexol concentrations from blood samples, which produced a systematic overestimation of GFR by approximately 10%. This measurement error in this laboratory was described in a publication in 2017[2]. We subsequently repeated the analyses in the *PLOS ONE* paper using recalibrated (corrected) iohexol GFR values provided here in an updated version of Table 1.

**Table 1:** Clinical characteristics of HIV-positive and HIV-negative participants.

Clinical characteristics		HIV-positive (n = 187)	HIV-negative (n = 98)	P value
Age, years, median (P_25_, P_75_)		49 (45, 53)	49 (45, 54)	0.58
Body mass index, kg/m^2^, median (P_25_, P_75_)		26 (23, 31)	27 (23, 33)	0.21
Sex	Female, n (%)	66 (35)	18 (18)	0.0027
	Male, n (%)	121 (65)	80 (82)	
Race	White, n (%)	11 (6)	8 (8)	0.46
	Black, n (%)	176 (94)	90 (92)	
Current smoker, n (%)		124 (66)	60 (61)	0.44
History of hypertension, n (%)		65 (35)	21 (21)	0.021
History of cardiovascular disease, n (%)		21 (11)	4 (4)	0.048
Hepatitis C seropositive, n (%)		100 (54)	28 (29)	0.0001
Systolic blood pressure, mm Hg, median (P_25_, P_75_)		120 (108, 131)	126 (113, 135)	0.0074
Diastolic blood pressure, mm Hg, median (P_25_, P_75_)		71 (65, 77)	73 (66, 82)	0.058
Glycosylated hemoglobin, %, median (P_25_, P_75_)		5.4 (5.1, 5.7)	5.5 (5.3, 5.8)	0.038
High-sensitivity C-reactive protein, mg/dL, median (P_25_, P_75_)		1.7 (0.6, 4.2)	1.9 (0.7, 5.5)	0.43
Percentage activated^a^ CD4 cells, median (P_25_, P_75_)		8.3 (5.4, 14.1)	3.8 (3.1–5.9)	<0.0001
Percentage activated^a^ CD8 cells, median (P_25_, P_75_)		30.7 (19.2, 46.9)	10.8 (7.7, 20.5)	<0.0001
Urine albumin-creatinine ratio, mg/g, median (P_25_, P_75_)		7 (3, 19)	5 (3,11)	0.18
Urine albumin-creatinine ratio > 30 mg/g, n (%)		36 (19)	9 (9)	0.027
Serum creatinine, mg/dL, median (P_25_, P_75_)		0.9 (0.8, 1.1)	1.0 (0.8, 1.1)	0.19
Serum cystatin C, mg/L, median (P_25_, P_75_)		0.93 (0.82,1.10)	0.84 (0.76, 1.10)	0.0002
Measured glomerular filtration rate, ml/min/1.73m^2^, median (P_25_, P_75_)		90 (76, 103)	97 (84, 111)	0.0044
eGFR_cr_, ml/min/1.73m^2^, median (P_25_, P_75_)		103 (85, 118)	103 (92, 114)	0.84
eGFR_cys_, ml/min/1.73m^2^, median (P_25_, P_75_)		87 (70,103)	101 (81, 112)	0.0001
eGFR_cr-cys_, ml/min/1.73m^2^, median (P_25_, P_75_)		95 (81, 109)	100 (89, 114)	0.012
Taking antiretroviral therapy, n (%)		171 (91)	-	-
Taking tenofovir, n (%)		127 (68)	-	-
Nadir CD4 count, cells/mm^3^, median (P_25_, P_75_)		145 (42, 301)	-	-
Current CD4 count, cells/mm^3^, median (P_25_, P_75_)		464 (248, 627)	-	-
HIV RNA > 400 copies/mL, n (%)		38 (20)	-	-
HIV RNA in subjects with values > 400 copies/mL, median (P_25_, P_75_)		11,680 (4,562, 62,084)	-	-

P_25_ and P_75_, 25^th^ and 75^th^ percentiles, respectively; eGFR_cr_, eGFR_cys_, and eGFR_cr-cys_ are glomerular filtration rates estimated by CKD-EPI equations using plasma creatinine, cystatin C, and both biomarkers, respectively.

^a^ Activated CD4 or CD8 T-cells defined as expressing both CD38 and HLA-DR surface markers

Because mGFR was recalibrated approximately 10% lower and mGFR was central to analyses, almost all estimates in Table 2, Table 3, and Table 4 have been revised, with substantive changes described below. We also revised all Figs 1–3, although the clinical inferences from the figures are unchanged.

**Fig 1:**
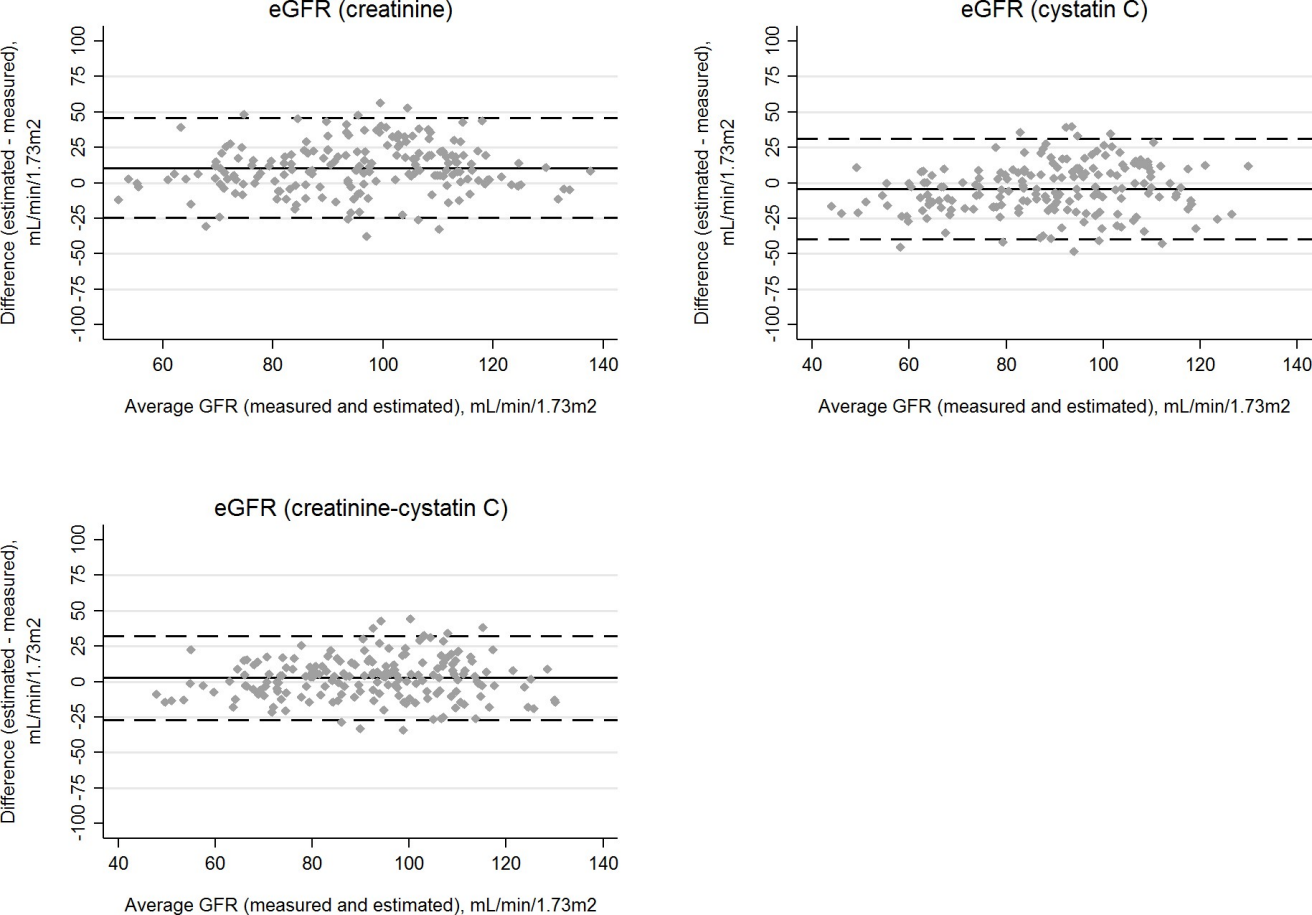
**Bland-Altman plots for estimated and measured glomerular filtration rate (GFR) in HIV-positive participants using the CKD-EPI equations for serum creatinine (A), cystatin C (B), or both biomarkers (C).** The average GFR (measured and estimated) is shown on the X axes. Bias, defined as the difference between estimated and measured GFR, is displayed on the Y axes. The average biases are represented by the horizontal solid lines and the horizontal dashed lines represent 2 standard deviations above and below the averages.

**Fig 2:**
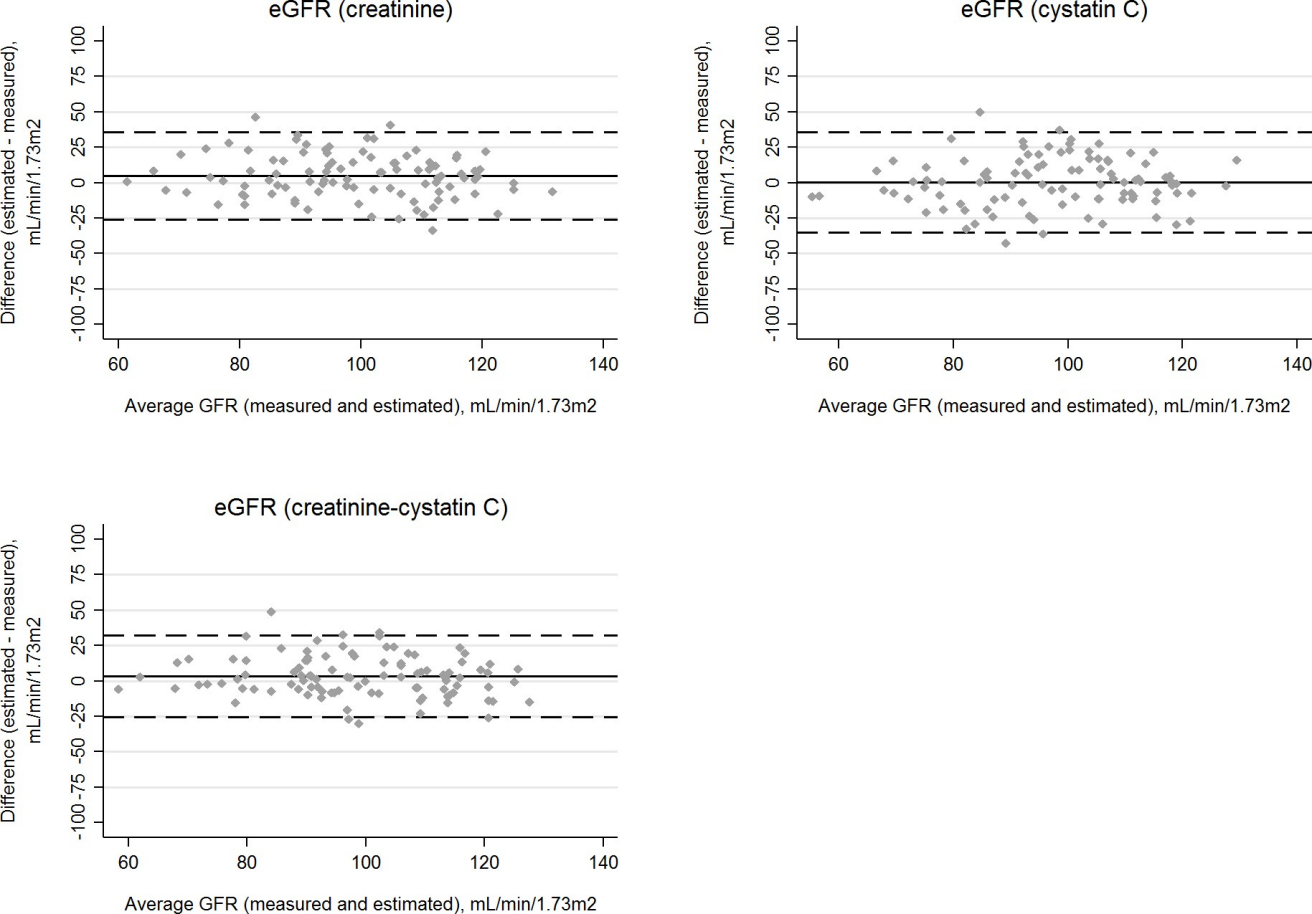
**Bland-Altman plots for estimated and measured glomerular filtration rate (GFR) in HIV-negative participants using the CKD-EPI equations for serum creatinine (A), cystatin C (B), or both biomarkers (C).** The average GFR (measured and estimated) is shown on the X axes. Bias, defined as the difference between estimated and measured GFR, is displayed on the Y axes. The average biases are represented by the horizontal solid lines and the horizontal dashed lines represent 2 standard deviations above and below the averages.

**Fig 3:**
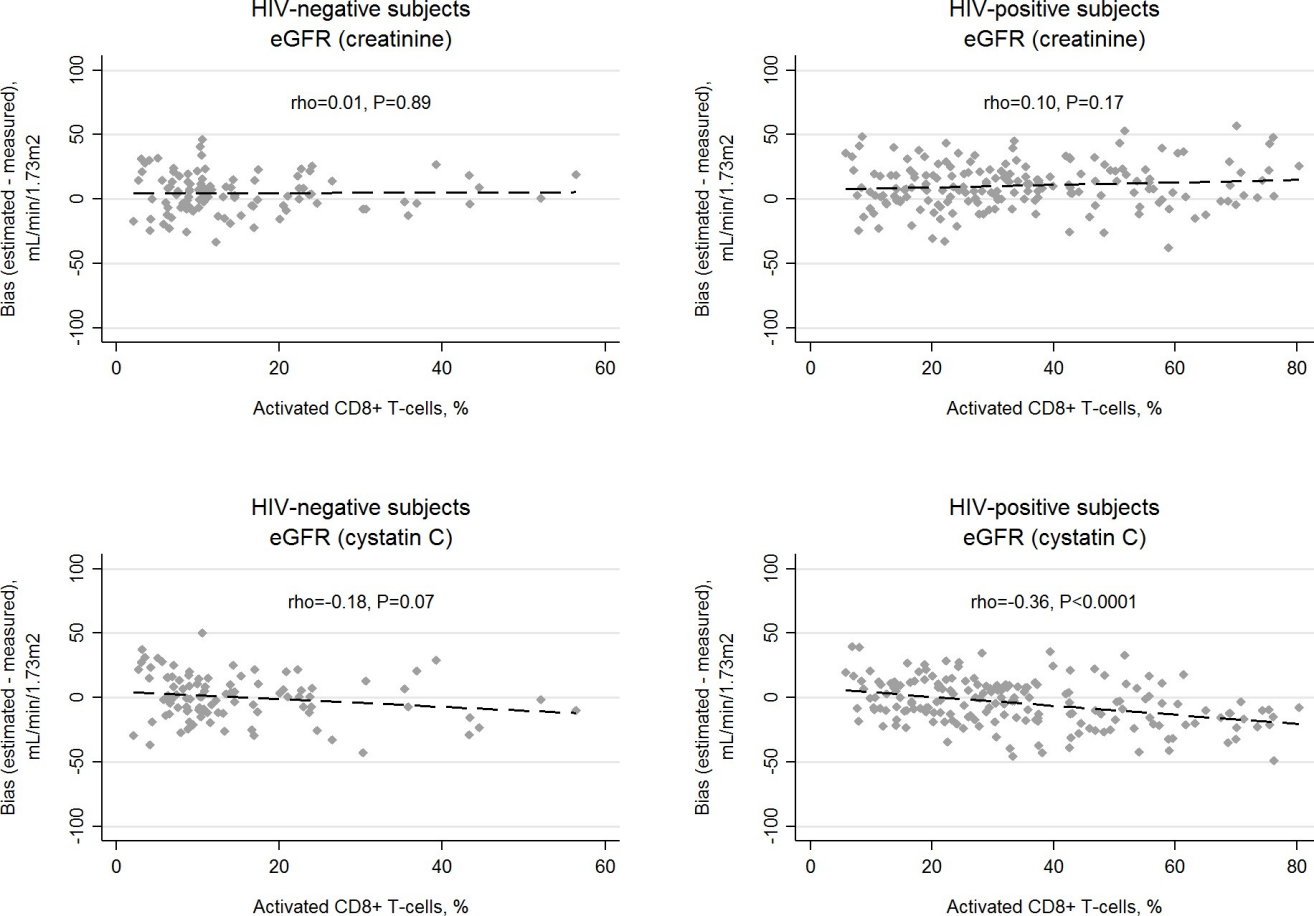
**Correlation of estimated glomerular filtration rate (eGFR) bias, defined as the difference between eGFR and measured GFR, with percentage of activated CD8 T cells (CD38+ and HLA-DR+) using the creatine-based CKD-EPI equation in HIV-negative (A) and HIV-positive (B) subjects, and the cystatin C-based CKD-EPI equation in HIV negative (C) and HIV-positive (D) subjects.** The percentage of CD8+ T cells with an activated phenotype is shown on the X axes (note, different scales for HIV-positive and HIV-negative groups). Rho is the spearman rank correlation coefficient, which may vary between -1 and 1. The dashed lines represent least-squares regression lines.

**Table 2:** Performance of glomerular filtration rate estimating equations in HIV-positive and HIV-negative participants.

Performance measure		HIV-positive	HIV-negative	P value^a^
Accuracy^b^ (95% CI)	eGFR_cr_	79 (72, 85)	88 (80, 94)	0.075
	eGFR_cys_	86 (81, 91)	88 (80, 94)	0.85
	eGFR_cr-cys_	91 (86, 95)	93 (86, 97)	0.82
P value^c^	eGFR_cr_ vs. eGFR_cys_	0.06329	1.00	-
	eGFR_cr_ vs. eGFR_cr-cys_	**0.000032**	0.05878	-
	eGFR_cys_ vs eGFR_cr-cys_	0.08326	0.0587	-
Bias^d^ (P_25_, P_75_)	eGFR_cr_	9.1 (-0.8, 21.0)	3.5 (-6.1, 14.7)	**0.00496**
	eGFR_cys_	-4.6 (-17.1, 8.3)	0.5 (-11.2, 13.3)	0.0404
	eGFR_cr-cys_	3.6 (-8.2, 12.2)	2.5 (-6.0, 13.3)	0.821
P value^c^	eGFR_cr_ vs. eGFR_cys_	**<0.0001**	0.01557	-
	eGFR_cr_ vs. eGFR_cr-cys_	**<0.0001**	0.242	-
	eGFR_cys_ vs eGFR_cr-cys_	**<0.0001**	**0.0002**	-
Precision^e^ (95% CI)	eGFR_cr_	22.8 (18.4, 27.3)	20.9 (15.1, 26.7)	0.50
	eGFR_cys_	25.9 (22.1, 29.7)	24.5 (18.9, 30.2)	0.61
	eGFR_cr-cys_	22.0 (18.1, 25.9)	19.8 (13.2, 26.4)	0.49
P value^c^	eGFR_cr_ vs. eGFR_cys_	0.43	0.65	-
	eGFR_cr_ vs. eGFR_cr-cys_	0.43	0.27	-
	eGFR_cys_ vs eGFR_cr-cys_	0.12	0.10	-

CI, confidence interval; eGFR_cr_, eGFR_cys_, and eGFR_cr-cys_ are glomerular filtration rates estimated by CKD-EPI equations using plasma creatinine, cystatin C, and both biomarkers, respectively; P_25_ and P_75_, 25^th^ and 75^th^ percentiles, respectively

^a^ Comparisons of a single equation between the HIV-positive and HIV-negative groups. P values in bold font indicate difference is statistically significant accounting for multiple comparisons (see text).

^b^ Accuracy defined as percentage of estimated GFR values within 30% of measured GFR.

^c^ Comparisons of a different equations within the HIV-positive or HIV-negative group. P values in bold font indicate difference is statistically significant accounting for multiple comparisons (see text).

^d^ Bias defined as difference between estimated GFR and measured GFR (mL/min/1.73m^2^).

^e^ Precision defined as interquartile range of bias.

**Table 3:** Factors associated with glomerular filtration rate estimating equation accuracy^a^ in HIV-positive and HIV-negative participants.

Factor		HIV-positive		HIV-negative	
		eGFR_cr_	eGFR_cys_	eGFR_cr_	eGFR_cys_
Age, years	≤ 49	78 (69, 86)	85 (76, 91)	83 (70, 92)	87 (74, 94)
	> 49	80 (70, 87)	88 (80, 94)	94 (82, 99)	89 (77, 96)
	P value^b^	1.00	0.67	0.13	0.76
Body mass index, kg/m^2^	≤ 26	76 (67, 84)	82 (73, 89)	93 (82, 98)	87 (73, 95)
	> 26	82 (72, 89)	91, 83, 96)	83 (71, 92)	89 (77, 96)
	P value^b^	0.37	0.13	0.22	0.77
Sex	Female	72 (60, 83)	86 (75, 93)	74 (49, 91)	89 (67, 99)
	Male	83 (75, 89)	87 (79, 82)	91 (83, 96)	88 (78, 94)
	P value^b^	0.13	0.82	0.050	1.00
mGFR, mL/min/1.73m^2^	< 90	65 (55, 75)	81 (71, 88)	68 (51, 82)	79 (63, 90)
	≥ 90	93 (86, 98)	92 (85, 97)	100 (94, 100)	93 (84, 98)
	P value^b^	**<0.0001**	0.030	**<0.0001**	0.054
Hepatitis C serostatus	Negative	76 (66, 85)	88 (79, 94)	87 (77, 94)	87 (77, 94)
	Positive	81 (72, 88)	85 (76, 91)	93 (76, 99)	93 (76, 99)
	P value^b^	0.47	0.67	0.50	0.50
High-sensitivity C-reactive protein, mg/dL	≤ 1.8	79 (70, 87)	88 (80, 94)	92 (80, 98)	90 (78, 97)
	> 1.8	79 (69, 87)	84 (75, 91)	84 (71, 93)	86 (73, 94)
	P value^b^	1.00	0.52	0.36	0.76
Percentage activated CD4 cells	≤ Median^c^	82 (72, 89)	92 (84, 97)	82 (69, 91)	82 (69, 91)
	> Median^c^	77 (70, 85)	81 (71, 89)	94 (83, 99)	94 (83, 99)
	P value^b^	0.46	0.047	0.12	0.12
Percentage activated CD8 cells	≤ Median^d^	82 (72, 89)	93 (86, 97)	82 (68, 91)	84 (70, 93)
	> Median^d^	77 (67, 85)	80 (70, 88)	94 (83, 99)	92 (81, 98)
	P value^b^	0.46	0.015	0.071	0.23
Taking antiretroviral therapy	Yes	78 (71, 84)	88 (82, 93)		
	No	94 (70, 100)	69 (41, 89)		
	P value^b^	0.20	0.016		
Nadir CD4, cells/ mm^3^	> 150	81(71, 89)	88 (79, 94)		
	≤ 150	77 (67, 85)	85 (77, 92)		
	P value^b^	0.59	0.67		
Current CD4, cells/mm^3^	> 450	84 (75, 90)	91 (83, 96)		
	≤ 450	74 (64, 83)	82 (72, 89)		
	P value^b^	0.15	0.13		
HIV RNA, copies/ml	≤ 400	80 (72, 86)	90 (84, 99)		
	> 400	76 (60, 89)	74 (57, 87)		
	P value^b^	0.66	0.047		

eGFR_cr_ and eGFR_cys_ are glomerular filtration rates estimated by CKD-EPI equations using plasma creatinine and cystatin C, respectively; mGFR, measured glomerular filtration rate by iohexol clearance.

^a^ Accuracy shown as percent of estimated GFR values within 30% of measured GFR values (95% confidence interval).

^b^ P values in bold font indicate difference is statistically significant accounting for multiple comparisons (see text).

^c^ Medians 8.3% and 3.8% in HIV-positive and HIV-negative groups, respectively.

^d^ Medians 30.7% and 10.7% in HIV-positive and HIV-negative groups, respectively.

**Table 4:** Factors associated with glomerular filtration rate equation bias^a^ in HIV-positive and HIV-negative participants.

Factor		HIV-positive		HIV-negative	
		eGFR_cr_	eGFR_cys_	eGFR_cr_	eGFR_cys_
Age, years	≤ 49	8.3 (-2.6, 22.5)	-4.4 (-16.2, 9.9)	3.3 (-6.5, 15.0)	0.7 (-7.4, 15.0)
	> 49	10.2 (2.2, 19.6)	-6.2 (-17.9, 7.0)	6.3 (-5.1, 13.9)	-4.4 (-12.9, 10.9)
	P value^b^	0.53	0.46	0.81	0.29
Body mass index, kg/ m^2^	≤ 26	8.0 (-1.1, 22.2)	-6.5 (-18.4, 8.2)	2.1 (-7.8, 13.7)	-0.5 (-9.5, 13.3)
	> 26	10.2 (0.2, 20.8)	-3.0 (-14.3, 9.7)	4.2 (-3.2, 15.3)	0.6 (-11.7, 12.9)
	P value^b^	0.93	0.36	0.15	0.89
Sex	Female	15.5 (4.3, 25.1)	-3.0 (-16.8, 8.0)	12.1 (2.4, 23.4)	0.5 (-9.3, 8.8)
	Male	6.9 (-1.9, 18.7)	-5.9 (-17.1, 9.8)	1.7 (-7.8, 13.6_	0.8 (-11.6, 15.1)
	P value^b^	**0.0042**	0.96	**0.0019**	0.94
mGFR, mL/min/1.73 m^2^	< 90	15.1 (2.7, 29.2)	0 (-13.4, 9.9)	13.4 (-1.9, 24.1)	8.1 (-3.1, 21.7)
	≥ 90	5.7 (-4.5, 17.1)	-9.5 (-20.9, 5.7)	1.0 (-7.8, 9.3)	-5.5 (-13.7, 4.7)
	P value^b^	**0.0001**	**0.0029**	**0.0005**	**0.0001**
Hepatitis C serostatus	Negative	9.1 (-1.1, 22.6)	2.9 (-11.6, 12.7)	3.4 (-6.3, 15.3)	3.5 (-7.4, 16.1)
	Positive	8.6 (0.9, 20.3)	-9.8 (-18.4, 4.4)	3.3 (-5.6, 12.7)	-9.5 (-21.4, -0.4)
	P value^b^	0.85	**0.0008**	0.91	**0.0003**
High-sensitivity C-reactive protein, mg/dl	≤ 1.8	9.1 (1.7, 21.0)	-3.4 (18.9, 8.1)	3.4 (-8.5, 14.4)	-0.5 (-11.5, 14.9)
	>1.8	8.6 (-1.1, 22.2)	-6.2 (-15.4, 9.2)	4.2 (-4.7, 15.3)	0.6 (-8.7, 9.1)
	P value^b^	0.90	0.80	0.43	0.76
Percentage activated CD4 cells	≤ Median^c^	8.7 (-1.9, 18.6)	2.9 (-9.9, 10.9)	3.3 (-6.6, 19.8)	1.3 (-10.1, 15.8)
	> Median^c^	10.2 (1.2, 22,8)	-12.2 (-22.7, 1.3)	3.8 (-3.6, 10.1)	-5.5 (-11.6, 7.6)
	P value^b^	0.25	**<0.0001**	0.86	0.10
Percentage activated CD8 cells	≤ Median^c^	7.0 (-2.1, 19.4)	0.1 (-10.6, 12.4)	6.3 (-6.3, 17.7)	0.7 (-10.1, 15.8)
	> Median^c^	13.0 (1.3, 22.6)	-9.9 (-21.2, 4.5)	2.1 (-5.0 10.1)	-0.6 (-11.6, 7.6)
	P value^b^	0.11	**0.0002**	0.35	0.23
Taking antiretroviral therapy	Yes	9.1 (-0.8, 21.4)	-3.3 (-15.6, 9.4)		
	No	9.9 (0.8, 21.3)	-16.2 (-28.9, -9.7)		
	P value^b^	0.89	**0.0022**		
Nadir CD4 count, cells/mm^3^	>150	5.9 (-5.0, 19.6)	-8.3 (-17.8, 8.0)		
	≤150	13.2 (3.7, 22.8)	-2.8 (-15.5, 8.4)		
	P value^b^	**0.0035**	0.15		
CD4 count, cells/mm^3^	> 450	8.6 (-1.3, 19.4)	-3.0 (-13.3, 9.8)		
	≤ 450	10.2 (1.9, 23.4)	-7.9 (-21.7, 7.6)		
	P value^b^	0.13	0.027		
HIV RNA, copies/ml	≤ 400	9.5 (-0.9, 20.4)	-0.8 (-12.7, 10.2)		
	> 400	7.9 (1.3, 25.4)	-16.8 (-31.3, -7.1)		
	P value^b^	0.89	**<0.0001**		

eGFR_cr_ and eGFR_cys_ are glomerular filtration rates estimated by CKD-EPI equations using plasma creatinine and cystatin C, respectively; mGFR, measured glomerular filtration rate by iohexol clearance.

^a^ Bias defined as median difference between estimated glomerular filtration rate (GFR) and measured GFR (25^th^ percentile, 75^th^ percentile)

^b^ P values in bold font indicate difference is statistically significant accounting for multiple comparisons (see text).

^c^ Medians 8.3% and 3.8% in HIV-positive and HIV-negative groups, respectively.

^d^ Medians 30.7% and 10.7% in HIV-positive and HIV-negative groups, respectively

1. In the original paper, we reported that the cystatin C-based equation (eGFR_cys_) was the least accurate and most biased of the three CKD-EPI equations in HIV-positive participants. In the revised analysis, we found that the creatinine-based equation (eGFR_cr_) was the least accurate and most biased of the three equations. This is relevant because eGFR_cr_ is the most commonly used equation in clinical practice. Consistent with the original analysis, the combined biomarker equation (eGFR_cr-cys_) remained the most accurate and least biased equation.
2. In contrast to the original analysis, we found that the accuracy and bias of eGFR_cr_ varied significantly by stratum of mGFR (<90 vs. ≥90 mL/min/1.73m^2^) in both the HIV-positive and HIV-negative groups, such that this equation was more biased and less accurate at lower levels of kidney function than at higher kidney function. This is important, because accurate GFR estimation may be more important at lower compared with higher levels of kidney function.
3. Consistent with the original analysis, we found that the bias of eGFR_cys_ was influenced by immune activation and HIV viremia, whereas eGFR_cr_ performance was not affected by these factors (Fig 3). However, in contrast to the original analysis, these factors were no longer statistically significantly associated with the accuracy of eGFR_cys_.

Please see the revised Figs 1–3 and revised Tables 2–4 here.

A member of *PLOS ONE*'s Editorial Board reviewed the new results and underlying data and confirmed that they support the overall conclusions reported in the article.

## Supporting information

S1 File Study Dataset. Clinical Variables eGFR and mGRF.
(DTA)
